# Facile solvothermal synthesis of nitrogen-doped SnO_2_ nanorods towards enhanced photocatalysis

**DOI:** 10.1039/d2ra04900g

**Published:** 2022-10-07

**Authors:** Runhua Liao, Jing Han, Zhongyan Chen, Jing Wang, Haoyue Wu, Shuangqiu Huang, Cheng Yan, Zhu Wang

**Affiliations:** School of Materials Science and Engineering, Jingdezhen Ceramic University Jingdezhen 333403 Jiangxi China; School of Chemistry, The University of Sydney Sydney 2006 Australia cyan003@e.ntu.edu.sg; Institute of Environmental Research at Greater Bay/Key Laboratory for Clean Energy and Materials/Key Laboratory for Water Quality and Conservation of the Pearl River Delta, Ministry of Education, Guangzhou University Guangzhou 510006 China wangzhu@gzhu.edu.cn

## Abstract

Heteroatom doping has proved to be one of the most effective approaches to further improve the photocatalytic activities of semiconducting oxides originating from the modulation of their electronic structures. Herein, nitrogen-doped SnO_2_ nanorods were synthesized *via* facile solvothermal processes using polyvinylpyrrolidone (PVP) as a dispersing agent and ammonium water as the N source, respectively. Compared with pure SnO_2_ sample, the as-synthesized nitrogen-doped SnO_2_ nanorods demonstrated enhanced photocatalytic performances, evaluated by the degradation of rhodamine B (RhB), revealing the effectiveness of nitrogen doping towards photocatalysis. In particular, the optimal photocatalyst (using 0.6 g PVP and 1 mL ammonia water) could achieve up to 86.23% pollutant removal efficiency under ultraviolet (UV) light irradiation within 150 min, showing 17.78% higher efficiency than pure SnO_2_. Detailed structural and spectroscopic characterization reveals the origin of activity enhancement of nitrogen-doping SnO_2_ in contrast with pure SnO_2_. Specifically, the bandgap and the morphologies of nitrogen-doped SnO_2_ have changed with more chemisorbed sites, which is supposed to result in the enhancement of photocatalytic efficiency. Moreover, the possible formation mechanism of nitrogen-doped SnO_2_ nanorods was discussed, in which PVP played a crucial role as the structure orientator.

## Abstract 2

Nitrogen-doped SnO_2_ nanorods were synthesized *via* facile solvothermal processes, which demonstrated enhanced photocatalytic performances evaluated by the degradation of rhodamine B (RhB), revealing the effectiveness of nitrogen doping towards photocatalysis.

## Graphical abstract

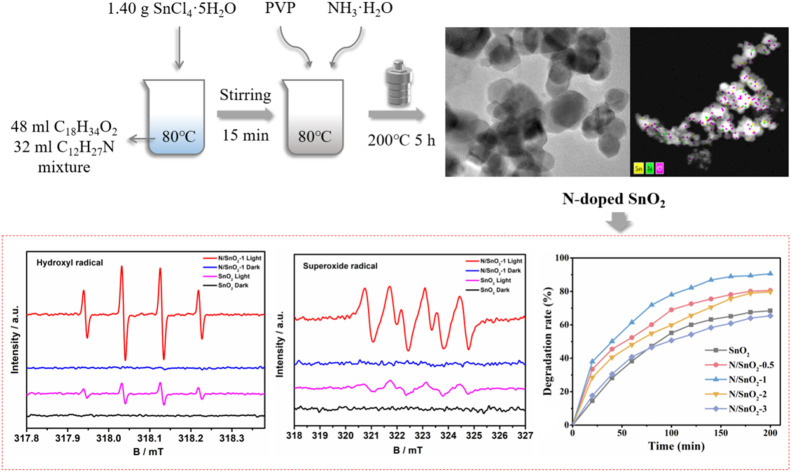

## 1. Introduction

The increasing demands for sustainable development ultimately require zero environmental pollution in the long run, including plastics, waste water, carbon dioxide and PM 2.5. However, pillar industrial manufacturing such as in the textile, food and cosmetics industries inevitably generates chemical pollutants and organic dyes,^1^ which are toxic and low-biodegradable, thus posing serious threats and hazard to the environment and living organisms. It is urgent and imperative to develop reliable strategies for the purification of industrial wastewater as well as the green removal of other pollutants. In the past decades, various kinds of treatment technologies to remove pollutants from waste water have been explored, aiming for ameliorating the pollution issues, including adsorption,^2^ Fenton-reagent method,^3,4^ membrane separation,^5^*etc.* Although tremendous progress has been made, almost all of them fail to completely destroy dye pollutants with low efficiency, require expensive equipment, and may lead to secondary pollution. In this context, emerging photocatalytic technology, which harvests the inexhaustible solar energy as the driving force, has been widely considered as one of the most promising sustainable pathways to address this challenge. Photodegradation capability of the specific oxide photocatalyst is mainly based on its oxidation behavior under the light irradiation. Various types of photocatalysts, including titanium dioxide (TiO_2_),^6,7^ zinc oxide (ZnO),^8,9^ tin oxide (SnO_2_),^10–12^ zirconium dioxide (ZrO_2_),^13^ and copper sulfide (CdS),^14,15^ have been extensively identified and developed in recent years. They can directly degrade organic pollutants into small molecules, such as H_2_O and CO_2_, and meet the stable, secure, green and environmental friendly needs.

Tin oxides, as a n-type semiconductor, have aroused widespread attention due to their low cost and environmental friendliness, are used in a variety of scenarios,^16,17^ such as gas sensors,^18–20^ lithium-ion batteries,^21^ solar cell,^22^ photocatalyst^23^ and so on. The wide band gap (3.6 eV), however, intrinsically limits the photodegradation of organic pollutants, owing to the low utilization efficiency of solar spectrum. More specifically, it can only capture the ultraviolet light with *λ* < 330 nm.^24^ In this regard, the construction of unique nano-morphology, the increase of specific surface area, active sites of catalytic reaction, and the transport of carriers to the surface of organic molecules by unique pore structure are the key factors to improve the photocatalytic efficiency. It turned out that the nanoporous SnO_2_ materials synthesized by photochemical route demonstrated higher degradation rate for methyl orange^25^ and rhodamine B.^26^ Moreover, porous SnO_2_ with hollow nanostructure synthesized by Liu *et al.*^27^ showed better photovoltaic performance due to their larger dye-absorbed effective surface area.

Heteroatom doping has proved to be one of the most effective approaches to further improve the photocatalytic activities of semiconducting oxides originating from modulation of electronic structures. In addition, previous studies showed that doped quantitative can effectively improve the crystallization properties of nanoparticles, control the grain size and change their properties. For example, Sato^28^ and Asahi^29^ found that the spectrum of the N-doped TiO_2_ was redshifted, which means it had better absorption performance under visible light. Early studies on N-doping showed that the valence band hybridization between nitrogen atom and semiconductor leads to the valence band electron delocalization of nitrogen atom and the upward shift of the valence band maximum.^30^ Since then, the researchers^31–34^ have found that nitrogen doping can improve its photocatalytic performance, because the ionic radius of nitrogen is relatively small (6% higher than that of oxygen) and has optimal electronic band position.^35^ On the other hand, the impurity level is introduced by N, so that the imaginary part of the dielectric function of the visible light region is increased, improving the absorption coefficient of the low energy region, making the absorption-edge red shift. Yan *et al.*^36^ predicted that N was a good p-type dopant source in theory. In addition, the N-doped model represented the p-type character and the red-shift phenomenon in the Sun's study.^37^ More specifically, the addition of PVP to tin dioxide powder could reduce the interfacial tension of the solution system and promote the formation of crystals. Moreover, nitrogen doping can facilitate the growth of tin dioxide lattices, which is beneficial to photocatalysis. In this regard, there are few studies reported for this experimental methodology combing nitrogen doping with tin oxides, both of which have their own advantages for the investigation of the photocatalytic properties.

On the basis of the above-mentioned discussion, the present work reported the facile synthesis of nitrogen-doped SnO_2_ nanocomposite by solvothermal method with PVP as dispersant and ammonia water as N source. Through adjusting the ratio of PVP to ammonia water, a series of samples have been successfully prepared. The structures and morphologies of as-synthesized SnO_2_ samples were investigated in detail. Compared with pure SnO_2_ sample, the as-synthesized nitrogen-doped SnO_2_ nanorods demonstrated enhanced photocatalytic performances evaluated by the degradation of rhodamine B (RhB), revealing the effectiveness of nitrogen doping towards photocatalysis. In particular, the optimal photocatalyst (0.6 g PVP and 1 mL ammonia water) could achieve up to 86.23% pollutant removal efficiency under ultraviolet (UV) light irradiation within 150 min, showing 17.78% higher than pure SnO_2_. Detailed structural and spectroscopic characterizations reveal that the origin of activity enhancement of nitrogen-doping SnO_2_ in contrast with pure SnO_2_. Specifically, bandgap and the morphologies of nitrogen-doped SnO_2_ have changed with more chemisorbed sites, which is supposed to result in the enhancement of photocatalytic efficiency. Moreover, the possible formation mechanism of nitrogen-doped SnO_2_ nanotubes was discussed, in which PVP played a crucial role as the structure orientator.

## 2. Experimental

### 2.1 Preparation of nitrogen-doped SnO_2_

**Fig. 1:**
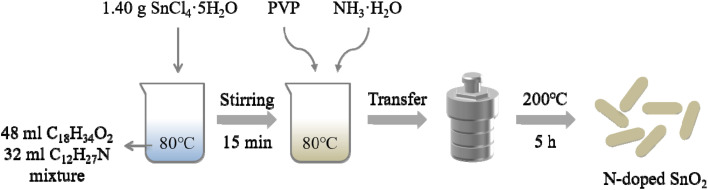
Experimental procedures for the synthesis of N-doped SnO_2_.

All reagents were of analytical purity (Guoyao Chemical Reagent Factory) and directly used as received. Firstly, 48 mL oleic acid (C_18_H_34_O_2_) and 32 mL twelve amine (C_12_H_27_N) were mixed in 150 mL beaker, and heated up to 80 °C. Subsequently, 1.40 g SnCl_4_·5H_2_O were dissolved in the mixed solution above and stirred for 15 min. The effects of dispersant PVP were studied by adding 0, 0.3 g, 0.6 g, 0.9 g and 1.2 g PVP, respectively; after pinpointing the optimal amount of PVP (0.6 g, see Results), a series of nitrogen-doped SnO_2_ samples were synthesized with the addition of 0 mL, 0.5 mL, 1 mL, 2 mL and 3 mL ammonia water to study the effect of nitrogen doping on the photocatalytic activities of tin oxides, and the corresponding samples were recorded as SnO_2_, N/SnO_2_-0.5, N/SnO_2_-1, N/SnO_2_-2, N/SnO_2_-3. All of the above were carried out under stirring with keeping the water bath temperature at 80 °C. After the solution was completely dissolved, transferred to a 100 mL PTFE container, and then put into a reaction kettle to react at 200 °C for 5 hours. When it is naturally cooling, the samples were washed several times with absolute ethanol. Finally, the sample was dried at 80 °C for 24 hours, then grind it into powder with the agate mortar. The experimental protocol for solvothermal synthesis of N-doped SnO_2_ is depicted as Fig. 1.

### 2.2 Photocatalytic activity

The photocatalytic activities of the as-synthesized samples were evaluated in RhB solution (100 mL, 50 mg L^−1^) as target compounds in wastewater under ultraviolet light irradiation. To establish adsorption–desorption equilibrium between photocatalysts and RhB solution, the RhB solution was continuously stirred in darkness for 30 min at first. Subsequently, the photocatalytic measurement was performed under a 1 kW mercury lamp using photochemical reaction instrument (BL-GHX-V, Shanghai billon Instrument Manufacturing Corporation). The samples were taken out from the reaction suspension before and after different reaction times, and the catalyst was removed by centrifugation at the speed of 4000 rpm for 2 min. The transparent solution was analyzed by a UV-vis spectrometer (A560, Shanghai Aoyi Instrument Corporation), and the absorbance was determined at a wavelength of 554 nm,^34^ which corresponds to the maximum absorption wavelength of RhB. The percentage efficiency of photodegradation of RhB solution was determined using following eqn (1).

1 *X* = (*C*_0_ − *C*)/*C*_0_ × 100%

where *C*_0_ refers to the initial concentration of RhB solution at the wavelength of 554 nm, and *C* represents the concentration of RhB solution after photocatalytic degradation for different time.

### 2.3 Characterization

The crystal structure and phase purity of the samples were examined by powder X-ray diffractometer (XRD, Bruker D8 Advance, Cu Kα radiation *λ* = 1.540 nm). The morphologies and structures were characterized by transmission electron microscopy (TEM, JEM-2010). X-ray photoelectron spectroscopy (XPS, THERMO SCIENTIFIC K-ALPHA) was utilized to analyze the valence. The Fourier transform infrared spectrometer (FT-IR Nicolet 5700), ranges from 400 to 4000 cm^−1^. The samples were tested with UV-vis diffuse reflectance spectroscopy (DRS, Lamda 850, United States) using an ultraviolet-visible light photometer. Porosity and Brunauer–Emmett–Teller^1^ surface areas of the products were evaluated by multi-point BET method with adsorption data. Electron paramagnetic resonance (EPR) spectra were performed using a Bruker ESR JES-FA200 spectrometer. The nitrogen adsorption–desorption isotherms were collected through a TriStar II 3020 Surface Area Analyzer. Transmission electron microscopy (TEM) images and high-resolution TEM image were obtained *via* a JEOL-2100 TEM with an acceleration voltage of 200 kV. UV-vis diffuse reflection spectra of the prepared samples were recorded on a UV-2450 UV-vis spectrophotometer.

## 3. Results and discussion

**Fig. 2:**
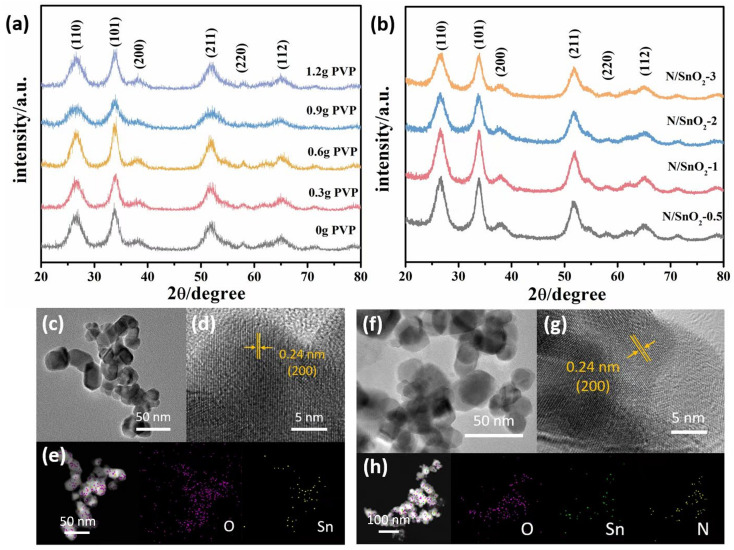
XRD patterns of (a) SnO_2_ with different amounts of PVP, (b) N/SnO_2_-0.5, N/SnO_2_-1, N/SnO_2_-2 and N/SnO_2_-3, (c) TEM, (d) HRTEM and (e) element mapping images of the as-prepared SnO_2_ with 0.6 g PVP, (f) TEM, (g) HRTEM and (h) element mapping images of the as-prepared N/SnO_2_-1.

Fig. 2a showed the XRD patterns of the as-prepared SnO_2_ samples with the addition of different amounts of PVP. It can be clearly observed in which the diffraction peaks could be observed at 2*θ* = 26.6°, 33.9°, 38.1°, 51.7°, 58.0° and 65.0°, corresponding to the (110), (101), (200), (211), (220), and (112) crystal planes, respectively, which were consistent with those of the standard card JPDS41-1445 with no other diffraction peaks were detected. The results showed that the synthesis purity was high and the crystal structure of prepared SnO_2_ was tetrahedral rutile phase. Moreover, it should be emphasized that with the increase of PVP, the diffraction peaks of (101) (110) (112) crystal planes became sharp at first, and then widened gradually, which indicated that it was adsorbed or wrapped by PVP molecule and limited the growth rate of crystal plane. Previous studies have demonstrated that concentrations of PVP in the reaction mixtures plays a key role in controlling of the synthesis of SnO_2_ in shape, because PVP contributes to the growth of oriented crystals.^38^ Therefore, it was concluded that PVP was added 0.6 g, the crystallization degree of SnO_2_ nanopowders was the highest.

As depicted in Fig. 2b, the diffraction peaks of all the XRD patterns of nitrogen-doped SnO_2_ samples were consistent with those of pure SnO_2_ (standard card JPDS41-1445), which indicated the high purity of the samples prepared in this work. Although the position of diffraction peak of SnO_2_ was not changed after doping nitrogen atom, but the intensity of diffraction peak on (110), (101), (112) plane was slightly enhanced and broadened. With the increase of nitrogen doping, the main peak surface increased at first and then weakened. When the volume of ammonia water was 1 mL, the peak shape of the sample was sharpest and the degree of crystallization was the best, demonstrating that nitrogen doping promoted the growth of SnO_2_ grain. Because the radius of N^3−^ was similar to that of O^2−^, the doped N element act on the SnO_2_ lattice, and the lattice parameters change, which affected the intensity of diffraction peak and the degree of crystallization.

TEM technique was performed to further examine the morphologies and structures of the as-synthesized samples. The as-prepared SnO_2_ with 0.6 g PVP showed the size of about 30 nm in length, width of 25 nm, and shape of a thin rod with relatively uniform distribution and no obvious agglomeration was observed (Fig. 2c). Moreover, the addition of PVP to tin dioxide powder, could reduce the interfacial tension of the solution system, decrease its size, and increase the specific surface area, indicating it can promote the formation of crystals and reduce the agglomeration of particles benefiting from the electrostatic effect and space location-obstruct effect. The surface of the crystal nucleus can be inhibited and the growth rate of the nucleus was reduced, thus reducing the occurrence of the agglomeration phenomenon.

Fig. 2f showed the TEM images of nitrogen-doped SnO_2_ samples prepared by adding 1 mL NH_3_·H_2_O. It can be clearly observed that the addition of ammonia water reducing the sizes compared with pure SnO_2_, which was consistent with the XRD results. Furthermore, it could be found that the addition of 1 mL ammonia water resulted in the nitrogen–SnO_2_ sample with a small, long and good shape and relatively uniform distribution and a small amount of agglomeration (Fig. 2f). However, with the increase amount of ammonia water, the morphology of the sample gradually changed from fine long stick to coarse short rod, which confirmed the addition of appropriate amount of ammonia was beneficial to the growth of SnO_2_ nanostructures.

Furthermore, Fig. 2d and g show the high resolution TEM (HRTEM) images of SnO_2_ and N/SnO_2_-1, respectively. Obviously, the high-resolution TEM image confirmed the lattice fringes with an interlayer distance of 0.24 nm, which is in accordance with (200) crystal planes of SnO_2_ and N/SnO_2_-1. The corresponding elemental mappings images of SnO_2_ and N/SnO_2_-1 (Fig. 2e and h) further proved that the nitrogen was doped into the tin oxide without changing its lattice, and we could observe the existence of N, O, Sn elements homogeneously distributed among the sample.

**Fig. 3:**
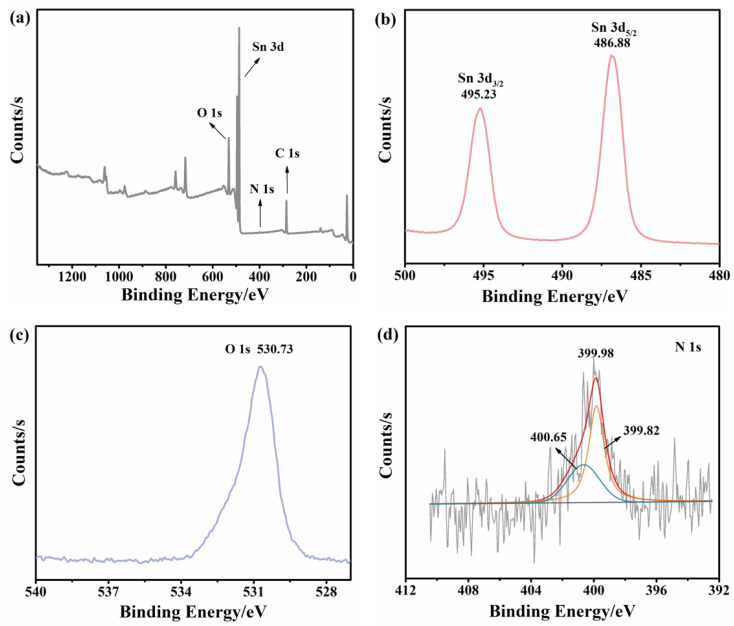
XPS spectra of N/SnO_2_-1 (a) full spectrum; (b) Sn 3d; (c) O 1s; (d) N 1s.

As a versatile tool for the analysis of chemical composition and valence states, X-ray photoelectron spectroscopy (XPS) was carried out to investigate the nitrogen-doped SnO_2_ samples. As shown in Fig. 3a, the full XPS spectrum of nitrogen-doped SnO_2_-1 confirmed the existence of Sn, O and N elements, matching well with the EDS results. Fig. 3b presented the high-resolution Sn 3d XPS with two distinct signal peaks at 495.28 and 486.88 eV corresponding to Sn 3d_5/2_ and Sn 3d_3/2_, respectively. Therefore, the formation of the Sn–O bond (486.6 ± 0.2 eV) could be identified^39^ and the spin–orbit splitting interval between the two characteristic peaks (8.5 eV) indicated the existence of Sn^4+^.^40^ The peak observed at 530.73 eV could be assigned to O 1s (Fig. 3c). As can be seen from Fig. 3d, the peaks located at around 400 eV were reported to be N^0^ and N^+^, which was consistent with chemisorbed N_2_.^41^ Combined with previous studies, it was highly confirmed that the peak N^+^ corresponds to nitrogen in chemisorbed states.^42^

**Fig. 4:**
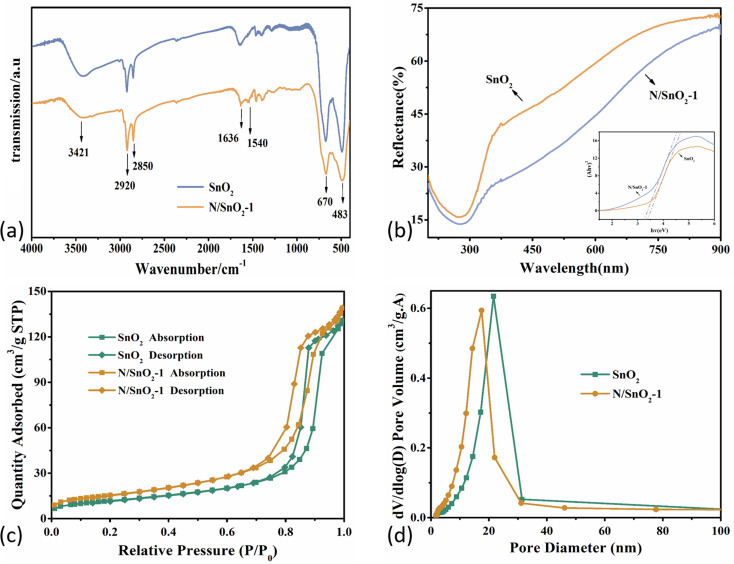
(a) FT-IR spectra; (b) UV-vis spectra; (c) N_2_ adsorption–desorption isotherms; (d) pore size distribution curve of the pure SnO_2_ and N-doped SnO_2_ samples.

Fig. 4a showed the FT-IR spectra of pure SnO_2_ and nitrogen-doped SnO_2_-1 samples. Specifically, the peaks centered at 670 cm^−1^ and 483 cm^−1^ were attributed to O–Sn–O and Sn–O.^43^ And the absorbing peaks in the range of 600–620 cm^−1^ can be assigned to the stretching vibration modes and bending vibration of Sn–O bond in SnO_2_,^44,45^ while the peaks at 2800–3000 cm^−1^ originated from CH_2_ and CH_3_,^46,47^ ascribed to organic species from the precursors during the heat treatment. Furthermore, the peak at 1540 cm^−1^ was attributed to N–H due to the addition of ammonia water, which indicated that the nitrogen atoms was doped into the tin dioxide, and some of the nitrogen was in the form of hydrogen bonds. The peaks at 1636 cm^−1^ and 3421 cm^−1^ corresponded to the stretching and bending vibration of H–O–H from H_2_O molecule absorbed by the SnO_2_ surface.^48^

Fig. 4b depicted the UV-vis reflectance spectra of the SnO_2_ and N/SnO_2_-1 samples. As is well known, the band gap of commercial SnO_2_ is about 3.6 eV. The energy (*E*_g_) band gaps can be determined based on Kubelka–Munk eqn (2):

2 (*αhν*)^1/2^ = *A*(*hν* − *E*_g_)

The band gaps of SnO_2_ and nitrogen-doped SnO_2_-1 samples were calculated to be 3.51 eV and 3.38 eV, respectively, which means the optical band gap decreased after nitrogen doping, leading to slight redshift of the optical response threshold.

The photocatalytic activities of photocatalysts are highly dependent on their physical and chemical properties, which specifically refers to the specific surface area, light absorbing capacity and photogenerated charge carrier separation efficiency. Brunauer–Emmett–Teller (BET, ASAP-2020) method can be utilized to estimate the BET specific surface area in relative pressure (*P*/*P*_0_) ranging from 0.05 to 0.25 by means of adsorption isotherms. Fig. 4c showed the nitrogen adsorption–desorption isotherms of SnO_2_ and N/SnO_2_-1 samples, both of which showed a typical IV type isotherm with various H1 hysteresis loop, indicating the presence of cylindrical mesoporous in the N/SnO_2_-1 sample.^36^

As shown in Fig. 4d, the pore size distribution curve of samples indicated their average pore diameters were about 18.3 nm and 14.5 nm, and the BET surface area of pure SnO_2_ and N/SnO_2_-1 samples were 42.5415 m^2^ g^−1^ and 55.2165 m^2^ g^−1^, respectively. As discussed above from TEM images in Fig. 2, the SnO_2_ grains have been refined due to the addition of ammonia, which are mostly related to changes in the specific surface area and pore size of the as-synthesized samples. However, combined with the TEM results, the nitrogen-doped SnO_2_ with obvious aggregation when the amount of ammonia water was higher.

**Fig. 5:**
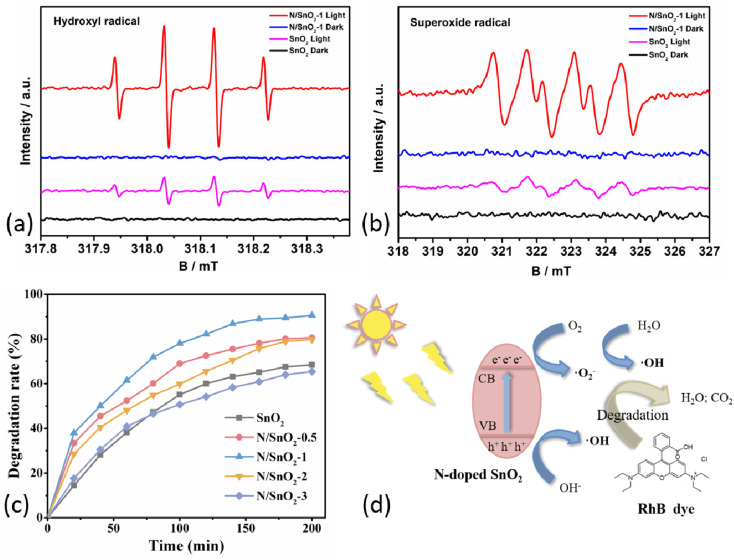
(a and b) EPR of samples, (c) the photocatalytic degradation rate of SnO_2_ sample under ultraviolet light, (d) mechanism diagram photocatalytic hydrogen production during organic pollutants photodegradation.

Electron paramagnetic resonance (EPR) analysis was demonstrated to show the charge transfer mechanism in SnO_2_ and N/SnO_2_-1 samples. Here, 5,5-dimethyl-1-pyrroline *N*-oxide (DMPO) was used as the trapping agent to capture the photoexcited active ·O_2_^−^ and ·OH. The DMPO–·O_2_^−^ and DMPO–·OH signal of all samples display no change under dark conditions (Fig. 5a and b). Where four peaks were observed for SnO_2_ and N/SnO_2_-1 in DMPO–·O_2_^−^, and the signal intensity of N/SnO_2_-1 was the stronger than that of SnO_2_. Which was identical to the results of all samples in DMPO–·OH. It also showed that nitrogen doping can improve the oxidation performance of SnO_2_ under ultraviolet light condition, corresponding to the photocatalytic degradation performance analyzed below. Namely, the photocatalytic activities of the as-prepared SnO_2_ samples were evaluated by photodegradation efficiency of RhB solution using ammonium hydroxide doped SnO_2_ under ultraviolet light irradiation. As shown in Fig. 5c and Table 1, the degradation efficiency of RhB was determined to be 68.45%, 80.55%, 86.23%, 79.78% and 65.44% under the same condition when the amount of ammonia water was 0, 0.5, 1, 2, and 3 mL, respectively. The improvement of the photocatalytic activity of nitrogen-doped SnO_2_-1 sample can be attributed to its unique microstructure. On one hand, the small particles can increase the active sites and help the electron–hole pairs transferring to the surface quickly, which can enhance the separation efficiency of the electron–hole pairs.^34^ On the other hand, it may be that nitrogen atoms entered the structure of SnO_2_ and formed more oxygen vacancy in order to make up for the charge imbalance, and the oxygen vacancy was the adsorption center of reactive oxygen species. The photocatalytic degradation mechanism of organic pollutants is illustrated in Fig. 5d. Reactive oxygen species can promote the effective separation of photoelectrons and holes, and accelerate the photocatalytic reactions, which collectively contributed to enhancement of photocatalytic performance of N-doped SnO_2_ sample. In addition, the grain surface area increased, and surfactant to the choice of different crystal had different adsorption, showing excellent adsorption performance. It was highly likely that the peak N^+^ corresponded to nitrogen in chemisorbed sites,^42^ which was consistent with the results mentioned above. Nevertheless, the excessive nitrogen doping will decrease the specific active surface area to some extent assuming that the active surface area of SnO_2_ is roughly constant. On the other hand, the addition of more ammonia water will lead to smaller particle size of the powder, which promotes the agglomeration phenomenon and reduces the specific surface area, lowering the photocatalytic efficiency accordingly.

**Table 1:** Comparison of rhodamine B dye removal efficiency of various photocatalysts

Material	Degradation rate of RhB
TiO_2_	68.45%^49^
C_3_N_4_	58%^50^
ZnO	79.93 (ref. 51)
CeO_2_	24.1%^52^
Ni–Al-LDHs	55.5%^52^
Mo/g-C_3_N_4_	57.6%^53^
g-C_3_N_4_	22%^53^
WO_3_/TiO_2_	50.7%^54^
SnO_2_	68.45% (in the study)
N/SnO_2_-1	86.23% (in the study)

## 4. Conclusions

In summary, nitrogen-doped SnO_2_ nanorods were successfully synthesized by facile solvothermal method with polyvinylpyrrolidone (PVP) K30 as the dispersing agent and ammonia water as nitrogen sources. It turned out that the sample prepared by the addition of 0.6 g PVP and 1 mL ammonia water showed the morphology of thin SnO_2_ slender rod with no obvious agglomeration. A possible mechanism for the formation of SnO_2_ was tentatively proposed, in which PVP played a key role as a structure-directing agent. Furthermore, the ammonia-doped tin dioxides changed the morphology and size but remained chemically. It can be concluded that nitrogen doping indeed promoted the photocatalytic activities of SnO_2_ nano-composite, reflected by the photo degradation efficiency of RhB solution. The degradation rate of RhB of nitrogen-doped SnO_2_-1 sample is up to 86.23% under ultraviolet light irradiation within 150 min, achieving 12.7% higher than pure SnO_2_ counterpart. This could be attributed to the peak N^+^ corresponded to nitrogen in chemisorbed sites. This work further demonstrated potential application value in energy conversion and environmental protection.

## Conflicts of interest

There are no conflicts to declare.

## Supplementary Material
